# Mutational signature of mtDNA confers mechanistic insight into oxidative metabolism remodeling in colorectal cancer

**DOI:** 10.7150/thno.78718

**Published:** 2023-01-01

**Authors:** Wenjie Guo, Yang Liu, Xiaoying Ji, Shanshan Guo, Fanfan Xie, Yanxing Chen, Kaixiang Zhou, Huanqin Zhang, Fan Peng, Dan Wu, Zhenni Wang, Xu Guo, Qi zhao, Xiwen Gu, Jinliang Xing

**Affiliations:** 1 State Key Laboratory of Cancer Biology and Department of Physiology and Pathophysiology, Fourth Military Medical University, Xi'an, China.; 2 State Key Laboratory of Oncology in South China, Collaborative Innovation Center for Cancer Medicine, Sun Yat-sen University Cancer Center, Sun Yat-sen University, Guangzhou, China.; 3 State Key Laboratory of Cancer Biology and Department of Pathology, Xijing Hospital and School of Basic Medicine, Fourth Military Medical University, Xi'an, China.

**Keywords:** mitochondrial DNA, somatic mutations, evolutionary selection, metabolic remodeling, colorectal cancer

## Abstract

**Rationale:** Mitochondrial dysfunction caused by mitochondrial DNA (mtDNA) mutations and subsequent metabolic defects are closely involved in tumorigenesis and progression in a cancer-type specific manner. To date, the mutational pattern of mtDNA somatic mutations in colorectal cancer (CRC) tissues and its clinical implication are still not completely clear.

**Methods:** In the present study, we generated a large mtDNA somatic mutation dataset from three CRC cohorts (432, 1,015, and 845 patients, respectively) and then most comprehensively characterized the CRC-specific evolutionary pattern and its clinical implication.

**Results:** Our results showed that the mtDNA control region (mtCTR) with a high mutation density exhibited a distinct mutation spectrum characterizing a high enrichment of L-strand C > T mutations, which was contrary to the H-strand C > T mutational bias observed in the mtDNA coding region (mtCDR) (*P* < 0.001). Further analysis clearly confirmed the relaxed evolutionary selection of mtCTR mutations, which was mainly characterized by the similar distribution of hypervariable region (HVS) and non-HVS mutation density. Moreover, significant negative selection was identified in mutations of mtDNA complex V (ATP6/ATP8) and tRNA loop regions. Although our data showed that oxidative metabolism was commonly increased in CRC cells, mtDNA somatic mutations in CRC tissues were not closely associated with mitochondrial biogenesis, oxidative metabolism, and clinical progression, suggesting a cancer-type specific relationship between mtDNA mutations and mitochondrial metabolic functions in CRC cells.

**Conclusion:** Our study identified the CRC-specific evolutionary mode of mtDNA mutations, which is possibly matched to specific mitochondrial metabolic remodeling and confers new mechanic insight into CRC tumorigenesis.

## Introduction

Mitochondria, as double membrane-bound organelles, play central roles in cell metabolism 1. The human mtDNA is a 16,569 bp circular double-stranded DNA that harbors a control region (mtCTR) of 1,122 bp and 37 genes that encode 2 rRNA, 22 tRNA, and 13 proteins of oxidative phosphorylation 2. Each cell contains many copies of mtDNA (typically 100-10,000). Due to being devoid of protective histone and inefficient DNA repair system, mtDNA exhibits a significantly higher mutation rate than nuclear DNA 3, which often leads to the coexistence of both mutant and wildtype mtDNA, a phenomenon known as heteroplasmy 4. Considering the irreplaceable role of mitochondrial in cellular life activities, mitochondrial dysfunction, especially that caused by mtDNA mutations and subsequent metabolic defects, is implicated in diverse human aging-related and degenerative disorders 5, especially in cancers 1.

Since the first discovery of somatic mtDNA mutations in cancer cells in 1998 6, extensive studies have focused on the characterization and functional evaluation of somatic mtDNA alterations in cancers. Consistent with the high mtDNA mutation rate, several pan-cancer studies demonstrated that somatic mtDNA mutations were prevalent and possessed cancer-specific mutation patterns, which mainly resulted from various evolutionary selection (positive, relaxed, and negative selection) during tumor development and progression 7-9. In addition, cancer-type related variations in mtDNA copy number were also observed across different tumors 9, 10. Furthermore, amounting evidence supported that mtDNA alterations and associated mitochondrial dysregulation had the potential to impact the development and progression of tumor, possibly in a cancer-specific manner 11-15. For example, Ishikawa K et al. have reported that mtDNA *ND6* gene mutations (G13997A and 13885insC) contributed to tumor progression by enhancing the metastatic potential of tumor cells 11. Wang et al. have observed the decreased mtDNA content in ovarian cancer (OV), which may be an important genetic event in the progression of OV 13.

The pattern and evolutionary selection of somatic mtDNA mutations in colorectal cancer (CRC) remained poorly defined. Earlier studies on mtDNA mutations in CRC often focused on a fraction of mtDNA, mostly the D-loop region, thus providing only a partial picture of mtDNA mutations 16-19. Recently, several studies have investigated the mutation profiling of the whole mitochondrial genome across multiple cancers including CRC, but these pan-cancer analyses are more privileged to identify the shared mtDNA mutational and selection patterns in cancers 7-9. Meanwhile, these pan-cancer analyses unavoidably led to the spreading of research focus, rendering thorough characterization of CRC-specific mtDNA evolutionary patterns unfeasible. What's more, our recent study of the mtDNA control region has suggested that there may exist tumor-type specific evolutionary selection in the mtDNA control region (mtCTR), in which CRC exhibited a significantly relaxed selection 20. However, the complete pattern of CRC-specific evolutionary selection across the whole mitochondrial genome remains poorly known.

Furthermore, the role of somatic mtDNA mutations in CRC progression and metabolic remodeling remained to be determined. Several experimental studies have supported the functional roles of mtDNA alterations in CRC tumorigenesis 14, 21, 22. For instance, Smith et al. have reported that age-related mtDNA mutations resulted in defective mitochondrial oxidative phosphorylation (OXPHOS) and increased risk of intestinal tumors in a tumor-prone mouse model with an additional mtDNA mutator phenotype 22. Sun XC et al. have demonstrated that increased mitochondrial biogenesis and mtDNA content promoted the proliferation and metastasis of CRC cells both *in vitro* and *in vivo* 14. However, patient-based studies indicate that mtDNA somatic mutations are more likely to be “passengers” rather than “drivers” in CRC 23, 24. Therefore, it is of great necessity to comprehensively detect somatic mtDNA mutations with larger datasets and analyze their potential roles in metabolic remodeling in CRC.

To address these issues, we generated a large somatic mtDNA mutation dataset from two private CRC cohorts and one public CRC cohort, and comprehensively characterized the CRC-specific mutation and evolutionary patterns. We also comprehensively evaluated the association of somatic mtDNA mutations with mitochondrial biogenesis and clinical progression, which may confer new mechanic insight into CRC tumorigenesis.

## Materials and Methods

### Patient enrollment, sample collection, and cell line culture

Two large colorectal cancer (CRC) patient cohorts were separately enrolled from different geographical regions. Cohort 1 included 432 CRC patients from both Xijing and Tangdu Hospitals, affiliated to Fourth Military Medical University (FMMU) in Xi'an, Northwestern China. Cohort 2 included 1,015 CRC patients from Sun Yat-sen University Cancer Center in Guangzhou, Southern China. Patient inclusion criteria were as follows: (1) histopathologically diagnosed as CRC; (2) undergoing surgical resection; (3) no treatment before sampling; (4) no history of other malignancy. The clinical characteristics of the two cohorts are summarized in **Table S1**. The median follow-up time was 72.0 months (95% CI, 69.9 to 76.0) for cohort 1 and 68.8 months (95% CI, 67.7 to 70.4) for cohort 2. 64 patients in cohort 1 lost follow-up. The study protocol was approved by the Ethics Committee of the FMMU (KY20183331-1) and the Sun Yat-sen University Cancer Center (B2019-031-01). Written informed consent was obtained from each patient.

Paired tumor and adjacent non-tumor (para-tumor) tissue samples were collected from each patient. Fresh samples in cohort 1 were frozen in liquid nitrogen for further usage, while samples in cohort 2 were formalin-fixed and paraffin-embedded. Hematoxylin-eosin slide of each selected sample for DNA extraction and sequencing was carefully reviewed by two pathologists to confirm the cancer cell content of at least 70% in tumor tissues and no contamination of cancer cells in adjacent non-tumor tissues. In addition, normal human colorectal epithelial cell line HIEC and 11 CRC cell lines (including DLD1 and HT29) from the American Type Culture Collection (ATCC, Manassas, VA) were cultured in RPMI-1640 (Gibco, Thermo Fisher Scientific, Inc., Waltham, MA) or Dulbecco's modified Eagle's medium (DMEM, Gibco) medium, supplemented with 10% fetal bovine serum (Sangon Biotech, Shanghai, China) and 1% penicillin-streptomycin (Solarbio, Beijing, China).

In addition, to evaluate whether mtDNA mutation signatures may be affected by different sampling strategies (single-region and multi-region tissue sampling), multiregional tumor samples were also collected from 13 CRC patients of cohort 1, which were acquired at the center and edge with each at least 0.5 cm away from the others as previously described 25. In different tissue samples, the sampling number varies with tumor diameter (sample size = 163).

### DNA extraction, library construction, next-generation sequencing (NGS), and mutation calling

Genomic DNA was extracted from fresh tissue samples and cell lines using ENZA DNA Kit (Omega, USA) and from formalin-fixed paraffin-embedded (FFPE) samples using QIAamp DNA FFPE kit (Qiagen) according to the manufacturer's protocol. All the DNA samples were quantified with Qubit 3.0 (ThermoFisher). For cohort 1 and 12 cell lines, library preparation and capture-based mtDNA sequencing were performed as previously described 25, 26. Briefly, genomic DNA was randomly fragmented by a focused ultrasonicator (Scientz98, Ningbo, China), and then DNA fragments between 300 and 500 bp were selected, end-repaired, ligated with sequencing adapters, amplified, and captured with biotinylated mtDNA probes. The captured mtDNA libraries were sequenced on Illumina HiSeqXTen (Illumina) platform using paired-end (PE) of 150 bp. For cohort 2, the detailed protocol for library construction and whole exome sequencing (WES) with the WESplus gene panel, which is an upgraded version of the standard WES and can capture mitochondrial DNA (HaploX Biotechnology, Shenzhen, China), has been previously described 27. The sequencing in cohort 2 was performed on NovaSeq 6000 platform (Illumina) using PE 150. The mapping strategy of sequencing reads and the mtDNA mutation calling pipeline were carried out as described in our previous study 20. A series of filter conditions were used to call mtDNA mutations, including (i) at least three reads in each strand supporting the alternative allele, (ii) the total sequencing coverage ≥ 100×, (iii) removing heterogeneity sites in rCRS repeat regions (66-71, 303-311, 514-523, 12418-12425, 16184-16193). In this study, tumor somatic mutations were defined as variants with variant allele frequency (VAF) ≥ 1% in tumor tissues and VAF < 0.5% in paired adjacent non-tumor tissues. Detailed quality control information was summarized in **Table S2**.

### Public mtDNA mutation datasets

Public somatic mtDNA mutation datasets from whole genome sequencing (WGS) or WES data of human CRC (public CRC cohort) tumor tissues were directly downloaded from four publications 8, 9, 12, 28. Detailed information is provided in **Table S3**. In addition, somatic mtDNA mutation data from 117 patients with hepatocellular cancer (HCC cohort) and 49 patients with ovary cancer (OV cohort) described in our previous publication 20 were used for comparison of mutation distribution in HVS and non-HVS of the mtDNA control region (mtCTR). Germline mtDNA mutation data in the healthy human population, which contain 11,551 mtDNA single base substitutions, was extracted from the most updated Phylotree (Build 17, 18 Feb 2016) 29 based on the protocol as previously described 30.

### Functional annotation of mtDNA mutations

All mutations were annotated using ANNOVAR software 31. MitImpact 3.0 32 was used to evaluate the functional impact of nonsynonymous mutations in mtDNA coding regions, which were classified as high, medium, low, or neutral impact. Mutations in the tRNA coding regions were classified as benign or deleterious based on the method described by Kondrashov et al. 33.

### Estimation of mtDNA content

Relative mtDNA content in CRC tissue samples from cohort 1 and three cell lines (DLD1, HT29, and HIEC) was measured by a quantitative real-time PCR (qRT-PCR)-based method as previously described 34. In brief, the ratio of the mitochondrial NADH dehydrogenase1 gene (*MT-ND1*) in mtDNA to the single copy nuclear human globulin gene (*HGB*) was determined using standard curves. In comparison, relative mtDNA content in CRC tissue samples from cohort 2 was determined as previously described 27. Briefly, relative mtDNA content was calculated as the ratio of the number of sequencing reads mapping to the mtDNA to that mapping to the nuclear genome, corrected by a purity and ploidy correction factor.

### Gene expression analysis

RNA-seq count data of 118 CRC tissue samples with available mtDNA mutation data in the public CRC cohort were downloaded from the Broad GDAC Firehose and used for expression analysis of mtDNA coding genes. Additionally, RNA-seq count data from 383 CRC samples and 359 non-tumor colorectal samples were downloaded from the Cancer Genome Atlas (TCGA) and used to analyze the expression of *COX IV*,* HSP60*,* and TFAM* genes. All RNA-seq count data were preprocessed with RSEM from Illumina HiSeq RNASeqV2. By using the Benjamini-Hochberg method after normalizing gene expression changes with the DEseq2 R package, fold changes (FC) and corrected *P* values were calculated for multiple comparisons.

### Western blot and immunohistochemistry (IHC)

Western blot was performed using proteins extracted from 3 cultured cell lines (DLD1, HT29, and HIEC) as previously described 35. Band intensity was determined by Image J software (National Institutes of Health, MD). IHC staining for CRC tissues was performed as previously described 36. The expression level of targeted proteins was independently evaluated by two pathologists blinded to the clinical data according to the proportion and intensity of positive cells determined within five microscopic visual fields per slide (200-fold magnification). IHC was then scored (ranging from 0 to 12) by multiplying the percentage of positive cells by the intensity. All antibodies and working conditions are listed in **Table S4**.

### Fluorescence imaging of mitochondrial ATP and biomass

Fluorescence imaging for three cell lines was performed as we previously described 37 under an Olympus laser scanning confocal microscope (Tokyo, Japan). The fluorescent dye MitoTracker Green FM (Invitrogen, Carlsbad, CA, USA) was used to evaluate mitochondrial biomass. The fluorescent probe ATP-Red 1 (MedChem Express, NJ, USA) was used to detect mitochondrial ATP abundance as previously described 38. Image J software (National Institutes of Health, MD) was used to measure fluorescence intensity.

### Assessment of oxygen consumption rate (OCR)

OCR indicating cellular mitochondrial function was measured using the Seahorse XF24 Analyzer and Mito Stress Test Kit as previously described 39. In brief, cells were plated in XF24 cell culture microplate (Seahorse Bioscience) at a density of 4 × 10^4^ cells/well. After 3 days of culture, OCR was obtained under basal conditions, and upon sequential injection of 1 µM oligomycin (Oligo), 1 µM carbonyl cyanide 4-(trifluoromethoxy) phenylhydrazone (FCCP), 2 µM rotenone (R) and antimycin A (AA). Seahorse buffer consists of DMEM medium, phenol red, 25 mM glucose, 2 mM sodium pyruvate, and 2 mM glutamine. The OCR values were calculated after normalization of cell number. All experiments were performed in triplicate.

### Statistical analysis

Statistical analyses were performed using GraphPad Prism version 8.3.0 software (San Diego, CA, USA). The mutation density was calculated by dividing the mean number of mutations per sample by the length of the region (kb). A substitution type proportion is calculated by dividing the total number of mutations in a specific region by the number of mutations with that substitution type. The Mann-Whitney *U* test was used for comparisons between two groups with continuous variables. The Chi-square test was used for categorical variables. Spearman's rank correlation analyses were used to test correlations between measured variables. Kaplan-Meier survival curve was plotted and compared by log-rank test. All *P* values were two-tailed and reported using a statistically significant level of 0.05.

## Results

### Mutational patterns of mtDNA in CRC tissues from two private patient cohorts and one public cohort

Two large private CRC cohorts (432 patients in cohort 1 from Northwestern China and 1,015 patients in cohort 2 from Southern China), which were largely comparable in host characteristics of patients (**Table S1**), were enrolled to investigate the mutational pattern of mtDNA in CRC tissues. These two cohorts were separately sequenced and analyzed, which can enable cross-validation of mtDNA mutational characteristics. A public CRC cohort (845 patients, **Table S3**) was also assembled to further corroborate our findings. Considering the high heterogeneity of CRC tissues, we first evaluated the effect of two sampling strategies including single-region and multi-region tissue sampling on the mutational pattern of mtDNA (**Figure S1**). As expected, except for mutation number per patient, no obvious difference in mtDNA mutation signatures was found between the two sampling strategies, indicating the feasibility of single-region sampling for further mtDNA mutational pattern analysis.

**Figure 1:**
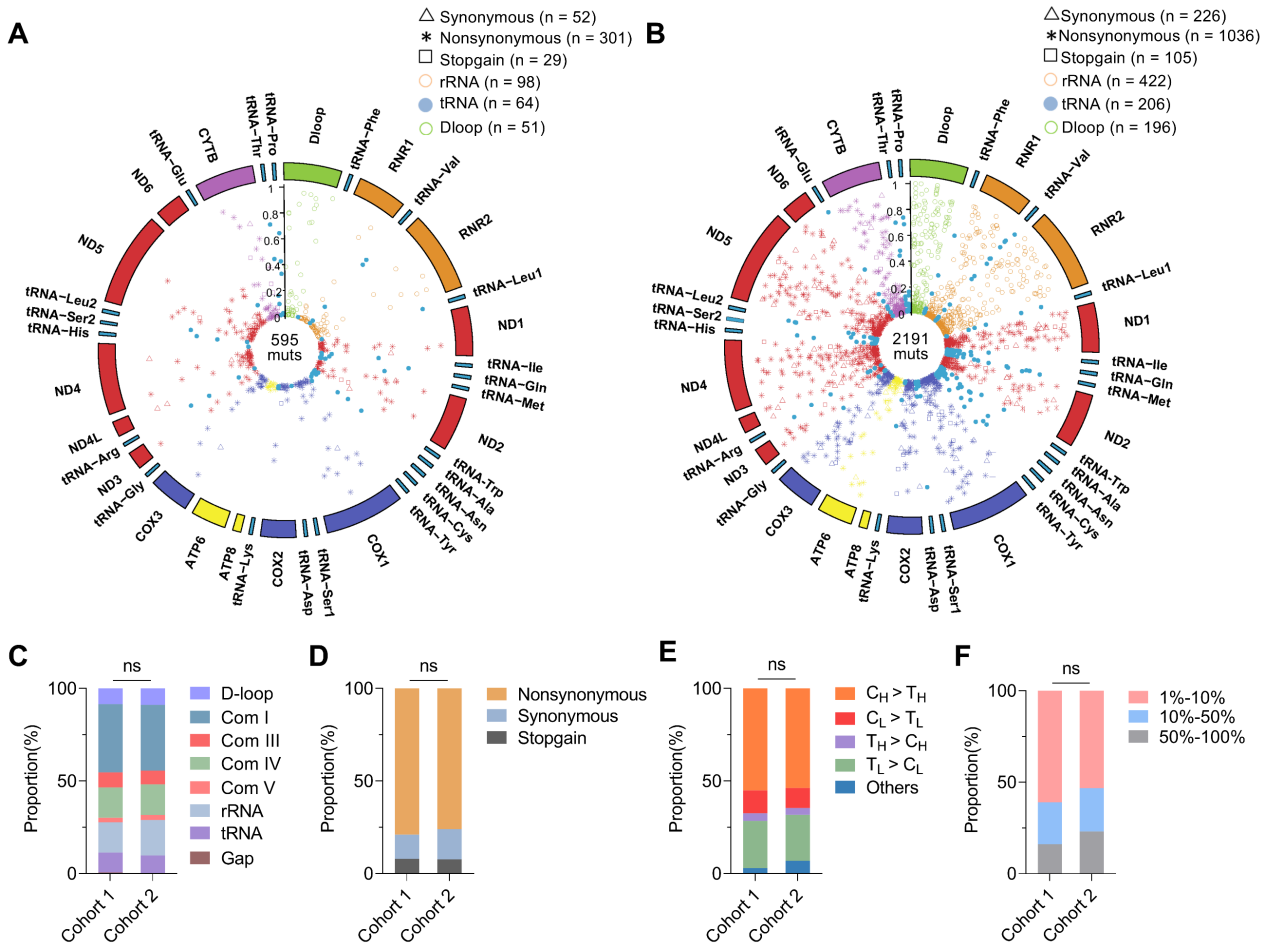
** Mutational pattern of mtDNA in CRC tissues from two patient cohorts. (A-B)** Circos plots showing the frequency and distribution of somatic mtDNA mutations from CRC patient cohorts 1 (n = 432) and 2 (n = 1,015). **(C)** The proportion of somatic mtDNA mutations in the functional regions of mtDNA, including D-loop, respiratory complexes (Com I, Com III, Com IV, and Com V), rRNA, and tRNA genes. **(D)** The proportion of synonymous, nonsynonymous, and stopgain mtDNA mutations. **(E)** The proportion of base substitution types for somatic mtDNA mutations. H for heavy strand, L for light strand, and other for all transversion mutations. **(F)** The proportion of three different heteroplasmic levels for somatic mtDNA mutations. Data were compared using Chi-square test.

Sequencing data of mtDNA were summarized in **Table S2**, with an average depth of 5,084 ± 2,357 × in cohort 1 and 9,668 ± 5,373 × in cohort 2. We identified 600 somatic mtDNA mutations in CRC cohort 1 (average 1.4 mutations per sample) and 2,200 somatic mtDNA mutations in CRC cohort 2 (average 2.2 mutations per sample). A complete catalog of the somatic mtDNA mutations in two cohorts was described in Circos plots (**Figure 1A-B**) and **Supplementary Data file**, revealing that the mtDNA mutations were distributed across the whole mitochondrial genome, with significant enrichment of mutations with low heteroplasmic levels. In addition, the two CRC cohorts exhibited similar distribution of somatic mtDNA mutations across different functional units of the mitochondrial genome (**Figure 1C**), similar predominance of nonsynonymous mutations (**Figure 1D**), similar dominance of transition over transversion mutations with strong H-strand (heavy-strand) C > T bias and L-strand (light-strand) T > C bias (**Figure 1E**), and similar proportions of variant heteroplasmic levels (**Figure 1F**). These observations were also confirmed in the public CRC cohort (data not shown). In addition, sequencing analysis of 11 CRC cell lines also identified similar distribution patterns of somatic mtDNA mutations across the mitochondrial genome (**Figure S2**).

### Region-specific evolutionary patterns of mtDNA mutations were shaped in CRC tissues

**Figure 2:**
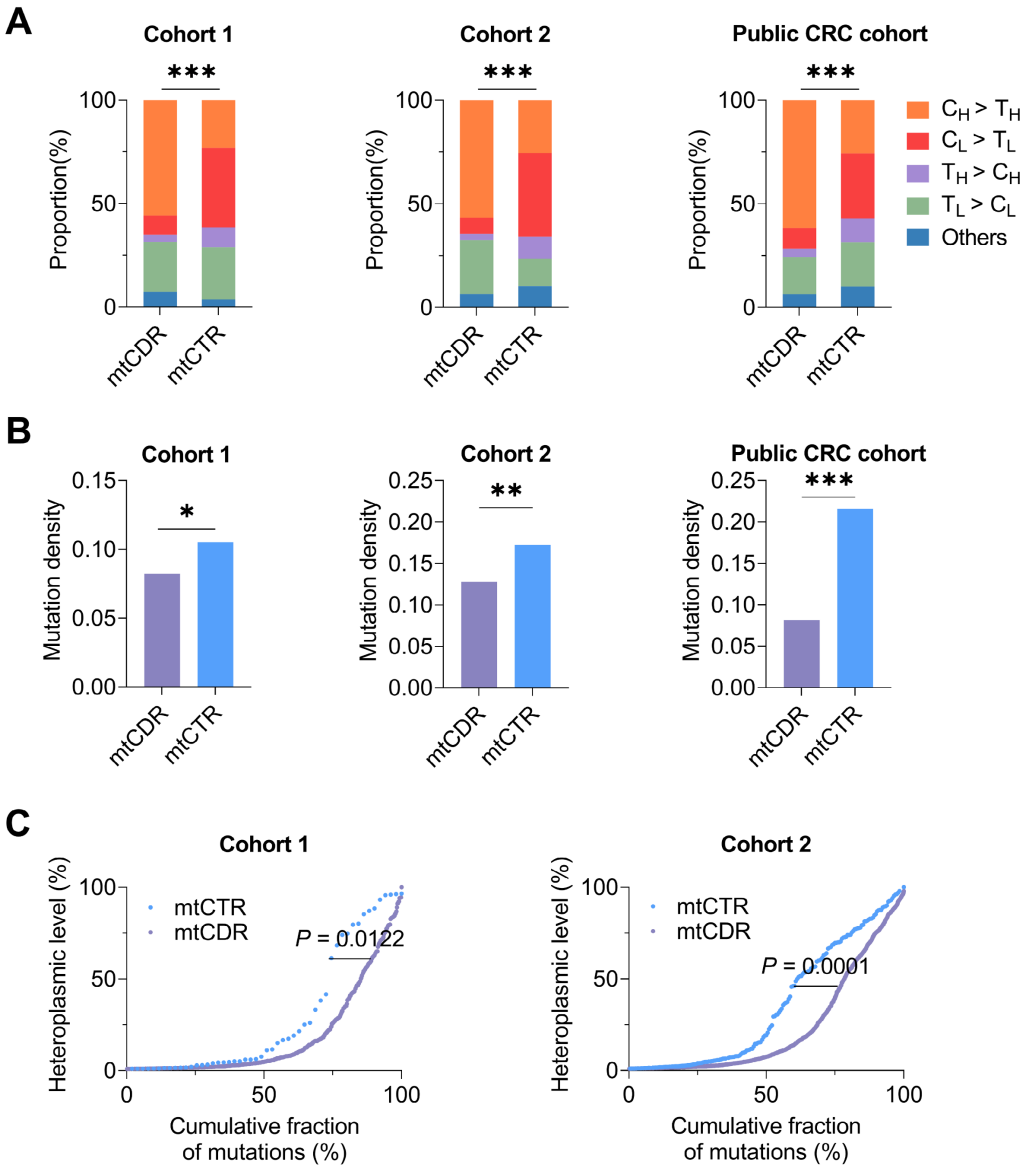
** Different characteristics of somatic mutations in mtDNA coding and control regions in three CRC cohorts. (A)** Proportion of base substitution types for somatic mutations in mtDNA coding (mtCDR) and control region (mtCTR) in CRC cohort 1, cohort 2, and public CRC cohort. **(B)** Mutation density for somatic mutations in mtCDR and mtCTR was calculated as the average number of mutations per sample per kilobase (kb). **(C)** Heteroplasmic level of accumulated mutations in mtCDR and mtCTR. Data were compared using Chi-square test **(A-B)** and Kolmogorov-Smirnov test **(C)**. ^✱^*P* < 0.05, ^✱✱^*P* < 0.01, ^✱✱✱^*P* < 0.001.

To explore the CRC-specific evolutionary pattern of mtDNA mutations, we first compared the mutational characteristics between two key functional mtDNA regions, including the control region (mtCTR) and coding region (mtCDR) based on to date the most comprehensive mtDNA somatic mutation dataset from three CRC cohorts. Our results showed that the mtCTR exhibited a distinct mutation spectrum characterizing a high enrichment of L-strand C > T mutations, which was contrary to the H-strand C > T mutational bias observed in the mtCDR (*P* < 0.001) (**Figure 2A**). The mtCTR also showed a significantly higher mutation density than the mtCDR (**Figure 2B**). Moreover, compared to mtCDR mutations, the mtCTR mutations exhibited a much more rapid accumulation of high heteroplasmic mutations (*P* < 0.05 in Cohort 1, *P* < 0.001 in Cohort 2) (**Figure 2C**). All these results strongly suggest that the mtCTR region may have a more relaxed evolutionary selection when compared to the mtCDR region.

### CRC exhibited a relaxed evolutionary selection of mutations in the mtDNA control region

**Figure 3:**
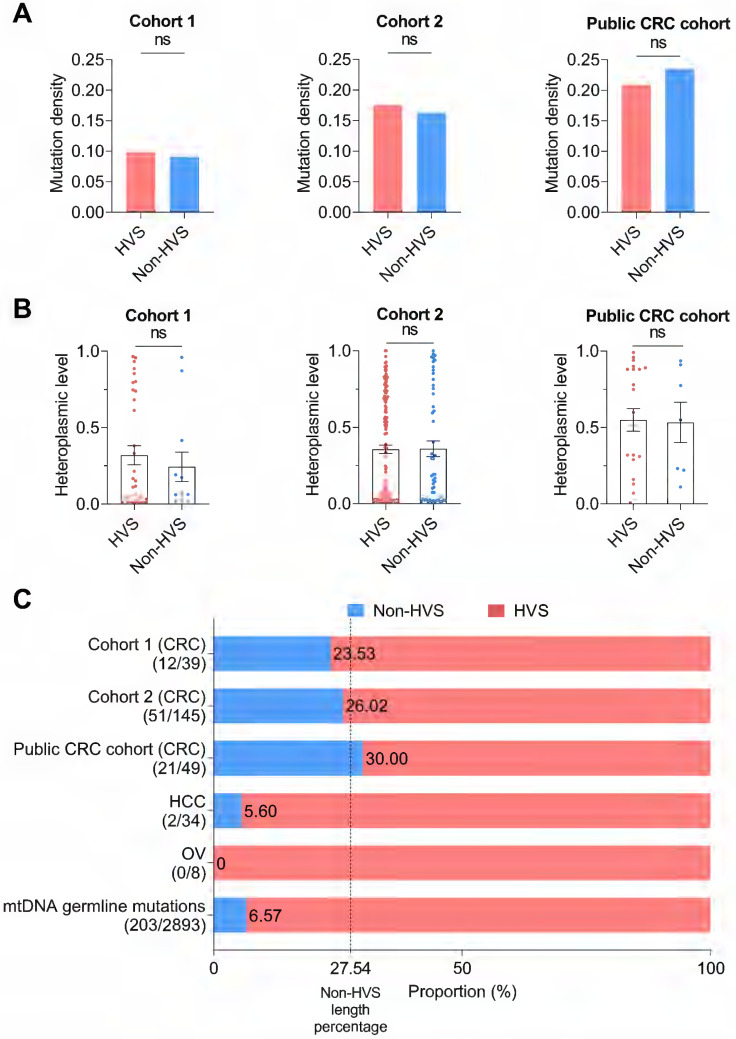
** Relaxed evolutionary selection of somatic mutations in the control region. (A)** Mutation density of the hypervariable (HVS) and non-hypervariable (non-HVS) mtDNA control region in CRC cohort 1, cohort 2, and public CRC cohort. **(B)** Heteroplasmic level of somatic mutations in the HVS and non-HVS mtDNA control region. **(C)** The proportion of somatic mutations in the HVS and non-HVS mtDNA control region for CRC cohorts, HCC cohort, OV cohort, and germline mutations retrieved from human mtDNA Phylotree (Build 17, 18 Feb 2016). The value 27.54 of vertical dashed line indicated the length percentage of non-HVS segment in mtCTR, which was used to represent possible percentage of non-HVS mutations in mtCTR mutations when evolutionary selection disappears. Mutation number in non-HVS and HVS regions was shown in brackets. HCC, hepatocellular cancer; OV, ovarian cancer. Data were compared using Chi-square test **(A)** and Mann-Whitney *U* test **(B)**.

The mtDNA control region (mtCTR), critical for the regulation of mtDNA replication and transcription, contains both hypervariable segments (HVS) and low-variable segments (non-HVS) 40. We thus compared the HVS and non-HVS mutations in mtCTR. Notably, we observed an elevated level of mutability in the non-HVS region in all three CRC cohorts, with both mutation density and levels of mutation heteroplasmy comparable to the adjacent HVS region (**Figure 3A-B**). Consistently, the relative proportion of HVS and non-HVS mutations exhibited a fairly good match with their respective length within mtCTR in all three CRC cohorts, which greatly contrasted to the relative shortage of non-HVS mutations in liver cancer, ovarian cancer, and also in germline mtDNA mutations (**Figure 3C**). Mutation distribution analysis further confirmed the lack of HVS and non-HVS mutational oscillation in all three CRC cohorts (**Figure S3**). Together, these data supported that the negative selection against non-HVS mutations was relaxed in colorectal cancer.

### Negative evolutionary selection of somatic mtDNA mutations in complex V (ATP 6/8) region in CRC tissues

**Figure 4:**
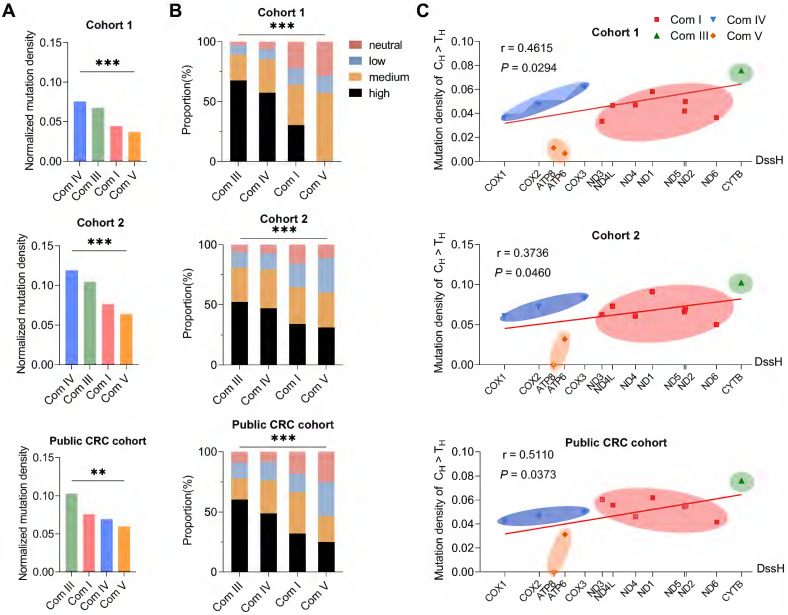
** Significant negative selection in mtDNA protein coding region.** The mutation density of C_H_ > T_H_ in Complex IV (the smallest in four complexes) was used as the standard. Mutation density of each complex was standardized based on the correlation of the mutation density of C_H_ > T_H_ with the DssH. Normalized mutation density was calculated as mutation density divided by the standardization score. **(A)** Normalized mutation density of mitochondrial respiration complexes (Com I, Com III, Com IV, and Com V) in CRC cohort 1, cohort 2, and public CRC cohort. **(B)** The proportion of pathogenicity for somatic mutations in mitochondrial complexes. **(C)** Gene-specific C_H_ > T_H_ mutation density based on DssH. Among them, *Cox1* has the lowest DssH value (0.11), while *Cytb* gene has the highest DssH value (1.15). The linear regression lines were also shown. Dssh, duration of mtDNA being single-stranded during replication. Data were compared using Chi-square test. ^✱^*P* < 0.05, ^✱✱^*P* < 0.01, ^✱✱✱^*P* < 0.001.

Next, we investigated the potential mode of evolutionary selection of somatic mutations in the four functional complexes (complexes I, III, IV, and V) consisting of 13 protein-coding genes of the mitochondrial genome. Interestingly, the mutation density of mtDNA coding regions at four mtDNA-encoded OXPHOS complexes differed significantly in all three cohorts (**Figure 4A**). Among them, complex V genes, including ATP 6 and ATP 8, showed the lowest mutation density (**Figure 4A**). In addition, variant pathogenicity analysis revealed a lower proportion of deleterious mutations in complex V genes (**Figure 4B**). In principle, the difference in mutation density can result either from different mutational rates or from different evolutionary selections. To distinguish these two possibilities, we evaluated the linear correlation between gene-specific mutation density and replication DssH (H-strand duration of being single-stranded during mtDNA replication), which can serve as a reliable estimation of relative mutation rate due to an intimate coupling of endogenous mtDNA mutagenesis and asymmetric mtDNA replication 30, 41. Indeed, H-strand C > T mutations (the predominant type of somatic mtDNA mutation) revealed an overall positive correlation between gene-specific mutation density and replication DssH time in all three CRC cohorts (r = 0.4615 and *P* = 0.0294 in Cohort 1; r = 0.3736 and *P* = 0.0460 in Cohort 2; r = 0.5110 and *P* = 0.0373 in public CRC cohort), supporting the presence of mutational gradient and the critical role of mtDNA replication for mtDNA mutagenesis in colorectal cancer (**Figure 4C**). In addition, consistent with the lowest mutation density for Complex V genes, we found that H-strand C > T mutations at the mitochondrial-encoded ATP synthase loci (ATP 6 and ATP 8) exhibited a downward deviation relative to its local DssH time in all three CRC cohorts (**Figure 4C**), supporting the existence of remarkable negative selection of ATP 6/ATP 8 mutations.

### Region-specific negative selection of mtDNA tRNA mutations in CRC tissues

**Figure 5:**
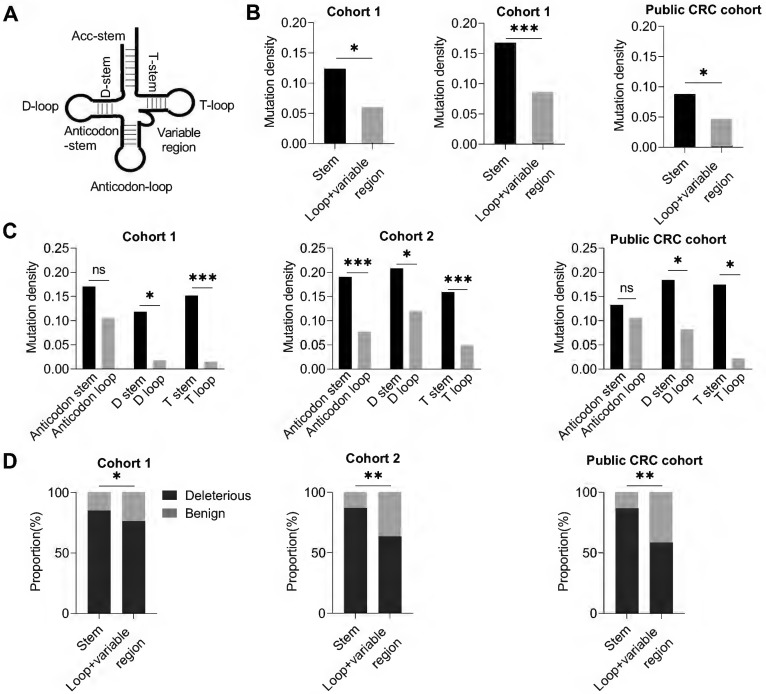
** Site-specific selection in mitochondrial tRNA region. (A)** Diagram of tRNA secondary structure. **(B)** Mutation density of mitochondrial tRNA stem region and non-stem region (loop and variable region) in CRC cohort 1, cohort 2, and public CRC cohort. **(C)** Mutation density of mitochondrial tRNA between the anticodon stem and anticodon loop, D stem and D loop, and T stem and T loop. **(D)** Proportions of deleterious and benign mutations in mitochondrial tRNA stem region and non-stem region. Data were compared using Chi-square test. ^✱^*P* < 0.05, ^✱✱^*P* < 0.01, ^✱✱✱^*P* < 0.001.

Further, we investigated whether somatic mutations in mitochondrial 22 tRNA-coding genes essential for mitochondrial protein translation were subjected to evolutionary selection in CRC tissues. Based on the secondary stem-loop structure (**Figure 5A**), tRNA was divided into two regions, the stem region, and the loop and variable region. Intriguingly, our data showed that the tRNA loop and variable region exhibited a significantly lower mutation density than the stem region in all three CRC cohorts (*P* < 0.05, *P* < 0.001, *P* < 0.05 in cohort 1, cohort 2, and public CRC cohort, respectively. **Figure 5B**). Further analysis revealed that all three major tRNA loop regions (D loop, anticodon loop, and T loop) exhibited a variable but consistent trend of decreased mutation density than their corresponding stem region (D stem, anticodon stem, and T stem) in all three CRC cohorts (**Figure 5C**). Moreover, the tRNA loop regions revealed a decreased proportion of deleterious mutations than tRNA stem regions in three CRC cohorts (**Figure 5D**). These data strongly suggest that the mitochondrial tRNA genes may also be subjected to region-specific evolutionary negative selection in CRC tissues.

Using the non-coding mtCTR region as a relaxed selection control, we also investigated the potential selection pattern of the mitochondrial rRNA genes (12S and 16S rRNA) in CRC tissues. Although the mutation density between mitochondrial rRNA and mtCTR region were comparable, the average heteroplasmic level of mutations in the rRNA coding region was significantly lower than that in the mtCTR in both cohort 1 and cohort 2 (**Figure S4**), suggesting that mtDNA rRNA mutations may also be subjected to negative evolutionary selection in CRC.

### Oxidative metabolism is commonly increased in CRC cells

**Figure 6:**
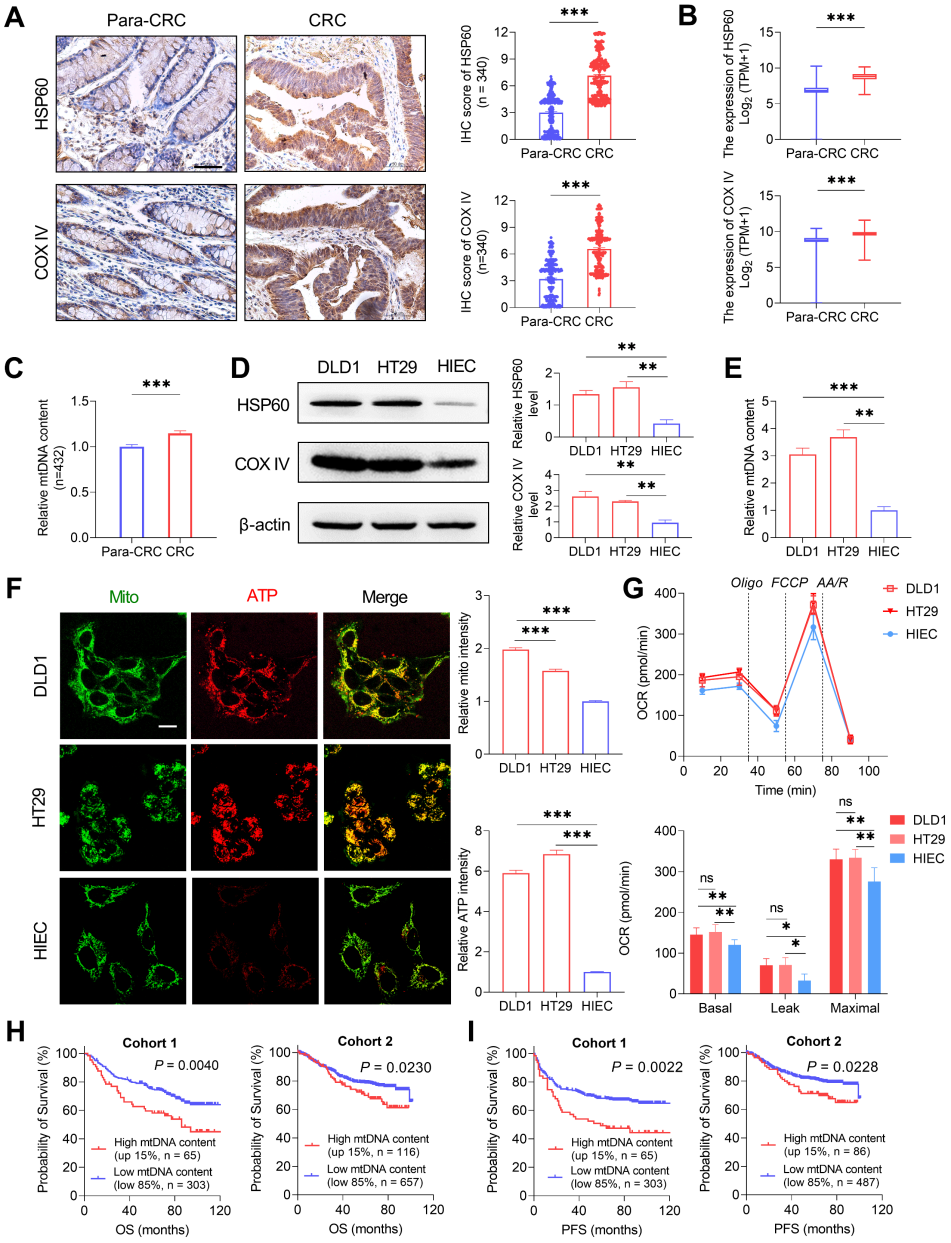
** Oxidative metabolism is commonly increased in CRC cells. (A)** Representative immunohistochemistry (IHC) staining and quantification of mitochondrial HSP60 and COX IV in CRC tissues (n = 340) and paired non-tumor tissues (Para-CRC, n = 340) random selected from CRC cohort 1. Scale bars: 50 μm. **(B)** Expression of *HSP60* and *COX IV* in CRC (n = 383) and para-CRC (n = 359) tissues based on RNA-seq counts retrieved from TCGA. **(C)** qPCR analyses of relative mtDNA content in paired CRC (n = 432) and Para-CRC (n = 432) tissues in cohort 1. **(D**-**E)** Western blot analyses **(D)** of HSP60 and COX IV and qPCR analyses **(E)** of relative mtDNA content in DLD1, HT29, and HIEC cell lines (n=3 independent experiments). **(F)** Representative confocal microscopy images and quantification of mitochondria ATP in DLD1, HT29, and HIEC cell lines. Scale bars: 15 μm. **(G)** Oxygen consumption rate (OCR) of mitochondria in normal human colorectal epithelial cell line HIEC and CRC cell lines DLD1 and HT29 was monitored by Seahorse analyzer (n = 3 independent experiments). **(H-I)** Kaplan-Meier curve analysis of overall survival (OS) and progression free survival (FPS) comparing CRC patients with a high tumor mtDNA content (top 15%) and those with a low tumor mtDNA content (bottom 85%) in CRC cohorts 1 and 2. In cohort 1, follow-up information was available for 368 patients. In cohort 2, mtDNA content information was available for 773 patients, in which progression free survival (PFS) information was available for 573 patients. Error bars represent mean ± SEM. Data were compared using the Mann-Whitney *U* test. ^✱^*P* < 0.05, ^✱✱^*P* < 0.01, ^✱✱✱^*P* < 0.001. Oligo, oligomycin; FCCP, carbonyl cyanide 4-(trifluoromethoxy) phenylhydrazone; AA/R, rotenone (R) and antimycin A (AA).

To investigate the possible functional roles of somatic mtDNA mutations in tumorigenesis of CRC, we first detected the mitochondrial oxidative metabolism in CRC tissues and cell lines. Both IHC and RNA-seq data analyses revealed that CRC tissues (from 340 CRC patients randomly selected from cohort 1) exhibited significantly increased expression of two representative mitochondrial biomass markers, mitochondrial matrix protein (HSP60, heat shock protein 60) and mitochondrial membrane-anchored respiratory chain component (COX IV) at both protein and mRNA levels when compared to the adjacent non-tumor tissues (**Figure 6A-B**). A significantly increased mtDNA content was also observed in CRC tissues than in adjacent non-tumor tissues in cohort 1 (**Figure 6C**). *In vitro* analysis also identified the higher expression of HSP60 and COX IV and higher mtDNA content in CRC cell lines (DLD1 and HT29) than in normal human colon cell line (HIEC) (**Figure 6D-E**). All these data indicate the increased mitochondrial biomass in CRC cells.

Furthermore, mitochondrial ATP abundance was determined by confocal microscopy with a mitochondria-targeted ATP probe. Our data revealed that the mitochondrial ATP abundance was significantly elevated in CRC cell lines (DLD1 and HT29) than in the normal colon cell line (HIEC) (**Figure 6F**). Consistent with this, Seahorse metabolic flux assay revealed the elevated level of mitochondrial respiration in CRC cell lines, with both basal oxygen consumption rate (OCR) and maximal OCR significantly higher than that of the normal HIEC cells (**Figure 6G**). In addition, survival analysis showed that patients with high tumor mtDNA content (top 15%) exhibited a significantly poorer prognosis than those with low mtDNA content (bottom 85%) in both CRC cohorts 1 and 2 (**Figure 6H-I**). These data suggest that mitochondrial oxidative metabolism may be commonly increased in CRC cells, supporting a critical role for mitochondrial oxidative metabolism in the tumorigenesis of colorectal cancer.

### mtDNA somatic mutations in CRC tissues are not closely involved in mitochondrial biogenesis and oxidative metabolism

**Figure 7:**
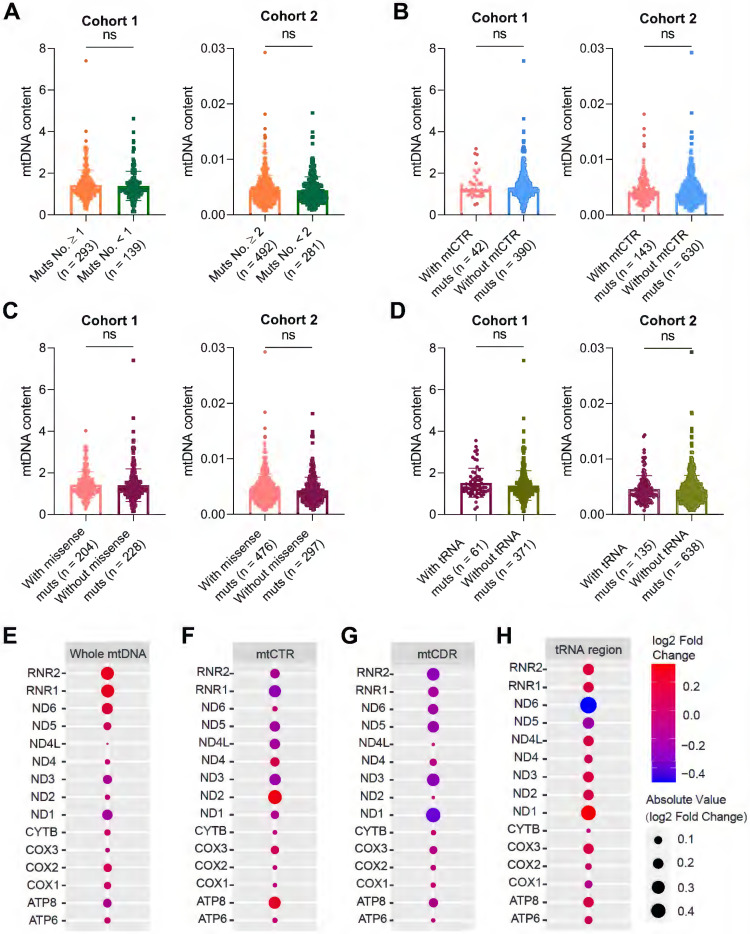
** mtDNA somatic mutations in CRC tissues are not associated with mitochondrial biogenesis and oxidative metabolic function.** Analysis of mtDNA content in CRC samples with a high or low number of mtDNA mutations **(A)**, with or without mtCTR mutations **(B)**, with or without missense mtCDR mutation **(C)**, and with or without tRNA mutations **(D)** in CRC cohorts 1 and 2. The median number of mtDNA mutations in cohorts 1 and 2 was used as threshold value to subgroup high or low number of mtDNA mutations, which was 1 in cohort 1 and 2 in cohort 2, respectively. **(E-H)** Analysis of mitochondrial gene expression between CRC samples with high (mtDNA mutation ≥ 3, n = 52) or low mtDNA mutation loads (mtDNA mutation < 3, n = 66) **(E)**, with (n = 28) or without (n = 90) mtCTR mutations** (F)**, with high (n = 52) or low (n = 66) number of missense mtCDR mutation** (G)**, and with (n = 26) or without (n = 92) tRNA mutations** (H)** in 118 TCGA CRC patients. The bubble colors correspond to Log2 fold change of gene expression level from high to low in two groups compared. *P* values were from the Mann-Whitney *U* test. mtCTR, mtDNA control region; mtCDR, mtDNA coding region; Muts, mtDNA mutations.

Given the critical role of mitochondrial oxidative metabolism, we next investigated whether somatic mtDNA mutations affect mitochondrial biogenesis in colorectal cancer. We found no significant difference in mtDNA content, a representative mitochondrial biomass biomarker, between CRC tissues with varying number of somatic mtDNA mutations (**Figure 7A**), CRC tissues with and without somatic mtCTR mutation (**Figure 7B**), CRC tissues with varying number of missense mtDNA mutations (**Figure 7C**), and CRC tissues with and without mtDNA tRNA mutations (**Figure 7D**). Consistently, the expression of two nuclear-encoded mitochondrial biogenesis biomarkers, HSP60 and COX IV, showed no significant difference between CRC tissues with high (Muts No. ≥ 1) and low (Muts No. < 1) mtDNA mutations in our cohort (**Figure S5A**). In contrast, the mtDNA content in CRC tissues was positively correlated with the expression of TFAM, the master mitochondrial transcription factor from the nuclear genome, which exhibited positive correlation with HSP60 at both protein and mRNA level (**Figure S5B-C**).

The expression of mitochondrial-encoded genes represented not only oxidative metabolism but also mitochondrial biogenesis. Transcription profiling of 15 mitochondrial encoded genes also found no significant difference between CRC tissues with high or low mtDNA mutation loads (**Figure 7E**), CRC tissues with and without somatic mtCTR mutations (**Figure 7F**), CRC tissues with varying number (more or less) of mtDNA protein-coding missense mutations (**Figure 7G**) and CRC tissues with and without mtDNA tRNA mutations (**Figure 7H**). These data supported that somatic mtDNA mutations were not associated with mitochondrial biogenesis and oxidative metabolism in CRC.

### mtDNA somatic mutations in CRC tissues are not associated with clinical progression

**Figure 8:**
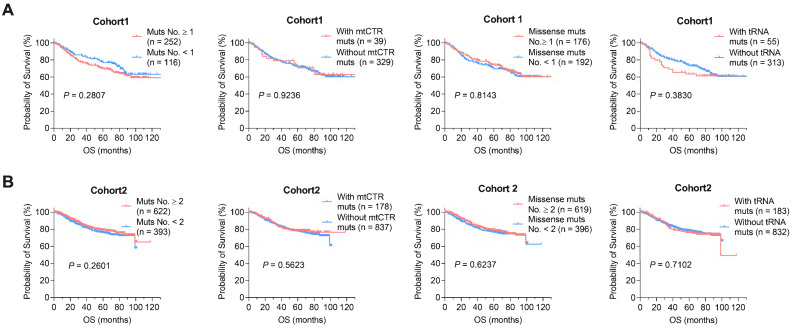
** mtDNA somatic mutations in CRC tissues are not associated with clinical progression. (A)** Kaplan-Meier curve analysis of overall survival (OS) between patients with different number of mtDNA mutations, mtCTR mutations, missense mtCDR mutations, and tRNA mutations in CRC cohort1.** (B)** Kaplan-Meier curve analysis of overall survival (OS) between patients with different number of mtDNA mutations, mtCTR mutations, missense mtCDR mutations, and tRNA mutations in CRC cohort 2. *P* values were calculated using the log-rank tests. Muts, mtDNA mutations.

We next analyzed whether somatic mtDNA mutations affect clinical progression of CRC. For this purpose, extensive Kaplan-Meier survival analysis identified no significant association between somatic mtDNA mutations and the overall survival (OS) of CRC patients in two CRC cohorts (**Figure 8A-B**). Also, we found no significant association between somatic mtDNA mutations and the progression free survival (PFS) of CRC patients (**Figure S6**). Together, these data suggest that the increased mitochondrial biogenesis may reprogram the CRC cells toward robust mitochondrial oxidative metabolism, which enables the toleration of somatic mtDNA mutations in CRC cells.

## Discussion

In this study, we generated to date the largest mtDNA somatic mutation dataset from three CRC cohorts and then most comprehensively characterized the CRC-specific evolutionary pattern. Moreover, two key findings were obtained. First, we clearly observed the relaxed selection of somatic mutations in the mtDNA control region, especially in the non-HVS region. Second, significant negative selection was identified in mutations of mtDNA complex V (ATP6/ATP8) and tRNA loop regions. We also showed that the CRC-specific evolutionary pattern of somatic mtDNA mutations was not associated with mitochondrial biogenesis and CRC patient prognosis, suggesting a cancer-type specific relationship between mtDNA mutations and oxidative metabolism remodeling in CRC cells.

Due to the rapid advance of next-generation sequencing technology, amounting studies have focused on the origin and functional consequence of mtDNA somatic mutations in tumorigenesis and metabolic remodeling. Based on mtDNA next-generation sequencing data across 31 tumor types, Ju et al. have carried out a comprehensive pan-cancer analysis, revealing mtDNA-specific mutation signatures (high transition dominance, high H-strand C > T, and L-strand T > C bias), which clearly support an mtDNA replication-coupled mutagenesis process 7. However, the huge intro- and inter-tumor heterogeneity combined with insufficient sample size have greatly limited the identification of cancer-type specific mtDNA mutation patterns in most tumors. Therefore, our recent study has analyzed somatic mtDNA mutations in hepatocellular carcinoma and identified significant positive selection of mtDNA control region mutations, which has relevance to mitochondrial biogenesis and HCC patient prognosis 15. In the present study, CRC-specific evolutionary mode of mtDNA mutations was identified, featuring a combination of relaxed selection of somatic mutations in the mtDNA control region and significant negative selection in mutations of mtDNA complex V (ATP6/ATP8) and tRNA loop regions, which is possibly matched to specific mitochondrial metabolic remodeling.

The mtDNA control region is divided into hypervariable segments (HVS 1-3) and less variable segments (non-HVS) 40. A pan-cancer analysis in our recent study has shown that the level and distribution of mtDNA control region mutations varied significantly across different cancer types; consequently, the relative distribution of HVS and non-HVS mutations defines different cancers into three types of evolutionary modes: relaxed, moderate, and strict constraint 20. In the present study, based on three CRC cohorts, we clearly confirmed that mtDNA control region mutations in colorectal cancer fit the relaxed selection mode, which is mainly characterized by a similar distribution of HVS and non-HVS mutations. Considering the critical role of mtCTR in mtDNA replication, we evaluated the association between mtCTR mutation and mtDNA copy number. Our data revealed that mtCTR mutations had no effect on mtDNA content in colorectal cancer. Meanwhile, we and others have found that CRC tissues exhibited an increased mtDNA content and increased mitochondrial biogenesis, which is associated with poor patient prognosis 42-44. We postulated that the increased mitochondrial biogenesis may enhance the mitochondrial robustness in colorectal cancer, which allows for a higher level of tolerance against mitochondrial control region mutations, especially non-HVS mutations.

Unlike the strong purifying selection in germline mtDNA mutations, previous pan-cancer analyses have generally identified an overall relaxed selection mode of mtDNA coding region mutations, mostly evidenced by the prevalence of nonsynonymous over synonymous mutations 7. In the present study, the high predominance of nonsynonymous mutations was also evident, supporting an overall relaxed selection of mtDNA coding region mutations in CRC. Importantly, our results revealed that mtDNA coding region mutations were subjected to region-specific selection, that is, negative selection against mtDNA complex V (ATP6/ATP8) and tRNA loop region mutations. These results suggest that maintenance of proper mitochondrial functionality, especially a functional protein translation and ATP generation system, may be required for colorectal cancer. Thus, the metabolic remodeling program in colorectal cancer may not be wired toward impairment of mitochondrial respiration. Consistent with this idea, a previous study has reported that overexpression of mitochondrial transcription factor A (TFAM) in CRC cells promotes the proliferation and metastasis of CRC cells via enhancing mitochondrial biogenesis and respiration 14. In the present study, we also observed the robust generation of mitochondrial ATP in CRC cell lines. In this context, the lack of association between mtDNA coding region mutations and CRC prognosis may simply suggest that these mutations do not have a major impact on mitochondrial respiration and thus are tolerated.

Consistent with our results showing increased mitochondrial biogenesis in CRC tissues and high oxidative metabolism in CRC cells, amounting studies have clearly shown that colorectal cancer cells, not all but at least a large part, are dependent on the high rate of OXPHOS 45, 46. To date, the exact mechanism underlying the mitochondrial metabolic remodeling in CRC cells remains to be explored. In the present study, we identified CRC-specific evolutionary selection of somatic mutations in mitochondrial control and coding regions, but found no significant impact of somatic mtDNA mutations on mitochondrial biogenesis and clinical prognosis. Our findings suggest that most somatic mtDNA mutations detected in CRC tissues may play a “passenger” role in CRC, with no evidence of positive selection. Accordingly, Tomasz G et al have reported that the presence of somatic mtDNA mutations was are not associated with any clinicopathological features in CRC, indicating that mtDNA somatic mutations are “passengers” rather than the cause of colorectal carcinogenesis 24. Meanwhile, Errichiello et al have reported that the homoplasmic mtDNA variants during tumor progression in CRC patients were most likely non-pathogenic (“passengers”), which are the most tolerable alterations for neoplastic cells 47. Considering that mitochondrial metabolic remodeling was under dual genetic control from both the nuclear and mitochondrial genomes, it is likely that nuclear-encoded factors play a more central role in the regulation of mitochondrial metabolism in CRC. Consistently, several nuclear-encoded factors, including PGC1a and TFAM, have been implicated in the control of mitochondrial biogenesis and OXPHOS in colorectal cancer 14, 48. Certainly, we cannot rule out the possibility of key mtDNA “driver” mutations in CRC, which may be missed in bulk mtDNA sequencing. In future studies, single-cell mtDNA sequencing can be applied for the identification of key mutations in cancers.

In combination, our study offers new insights into and provides a better understanding of CRC-specific mutation and evolutionary patterns, which is possibly matched to specific mitochondrial metabolic remodeling and confers new mechanic insight into CRC tumorigenesis.

## Supplementary Material

Supplementary figures and tables.

Supplementary data file.

## Abbreviations

CRC: colorectal cancer
DssH: H-strand duration of being single-stranded during mtDNA replication
FC: fold changes
FFPE: formalin-fixed, paraffin-embedded
HCC: hepatocellular cancer
HSP60: heat shock protein 60
HVS: hypervariable region
IHC: immunohistochemistry
mtDNA: mitochondrial DNA
mtCDR: mtDNA coding region
mtCTR: mtDNA control region
NGS: next-generation sequencing
OCR: oxygen consumption rate
OS: overall survival
OV: ovarian cancer
OXPHOS: oxidative phosphorylation
FPS: progression free survival
qRT-PCR: quantitative real-time PCR
TCGA: the Cancer Genome Atlas
TFAM: mitochondrial transcription factor A
VAF: variant allele frequency
WES: whole exome sequencing
WGS: whole genome sequencing
