# Supplementary material for: Mutational signature of mtDNA confers mechanistic insight into oxidative metabolism remodeling in colorectal cancer
Source: Theranostics. 2023 Jan 1;13(1):324–38. doi: 10.7150/thno.78718 (PMC9800724; doi:10.7150/thno.78718)
Supplement: Supplementary file 1 — Supplementary figures and tables. [file thnov13p0324s1.pdf]

Mutational signature of mtDNA confers mechanistic insight into oxidative metabolism remodeling in colorectal cancer

Wenjie Guo[1#], Yang Liu[1#], Xiaoying Ji[1#], Shanshan Guo[1], Fanfan Xie[1], Kaixiang Zhou[1], Huanqin Zhang[1], Fan Peng[1], Dan Wu[1], Zhenni Wang[1], Xu Guo[1], Qi zhao[2], YanXing Chen[2], Xiwen Gu[3*], Jinliang Xing[1*]

Supplementary materials include 6 figures and 4 tables:

Supplementary Figure 1. No obvious effect of the two sampling strategies on revealing mtDNA mutation signatures in CRC.

Supplementary Figure 2. Mutational pattern of mtDNA in 11 CRC and the HIEC control cell lines.

Supplementary Figure 3. Distribution of somatic mutations in three CRC cohorts and germline mutations in non-HVS and HVS of mtDNA control region (mtCTR).

Supplementary Figure 4. mtDNA rRNA mutations are subjected to negative evolutionary selection in CRC.

Supplementary Figure 5. Correlation between mtDNA mutations or TFAM expression and mitochondrial biogenesis biomarkers in CRC.

Supplementary Figure 6. mtDNA somatic mutations in CRC tissues are not associated with clinical progression.

Supplementary Table 1. Clinical characteristics of two private CRC cohorts.

Supplementary Table 2. Summary of mtDNA sequencing data from two private CRC cohorts.

Supplementary Table 3. Summary of mtDNA somatic mutations in public CRC cohort.

Supplementary Table 4. Primary antibodies used for Western blot and immunohistochemistry (IHC).

Supplementary figures

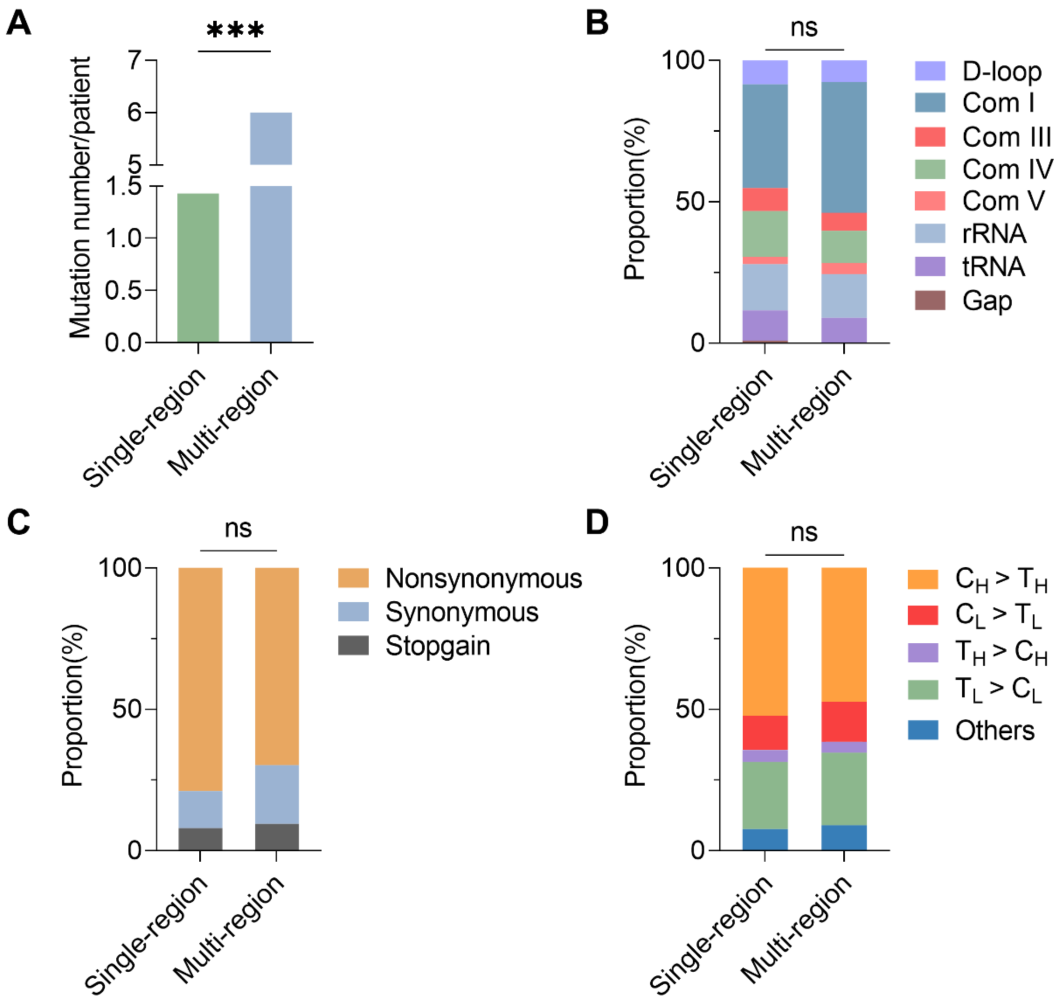

Supplementary Figure 1. No obvious effect of the two sampling strategies on revealing mtDNA mutation signatures in CRC. To evaluate the effect of different sampling strategies (single-region and multi-region tissue sampling) on revealing mtDNA mutation signatures in CRC, multiregional tumor samples were also collected from 13 CRC patients of cohort 1 (see details in methods). **(A-D)** Comparison of the number of mtDNA somatic mutations per sample **(A)**, the proportion of somatic mtDNA mutations in the functional regions of mtDNA, including D-loop, respiratory complexes (Com I, Com III, Com IV, Com V), rRNA and tRNA genes **(B)**, the

proportion of synonymous, nonsynonymous, and stopgain mtDNA mutations **(C)**, and proportion of base substitution types **(D)** for mtDNA somatic mutations detected by two sampling strategies. *P* values were from Mann Whitney *U* test **(A)** or Chi-square test **(B-D).**

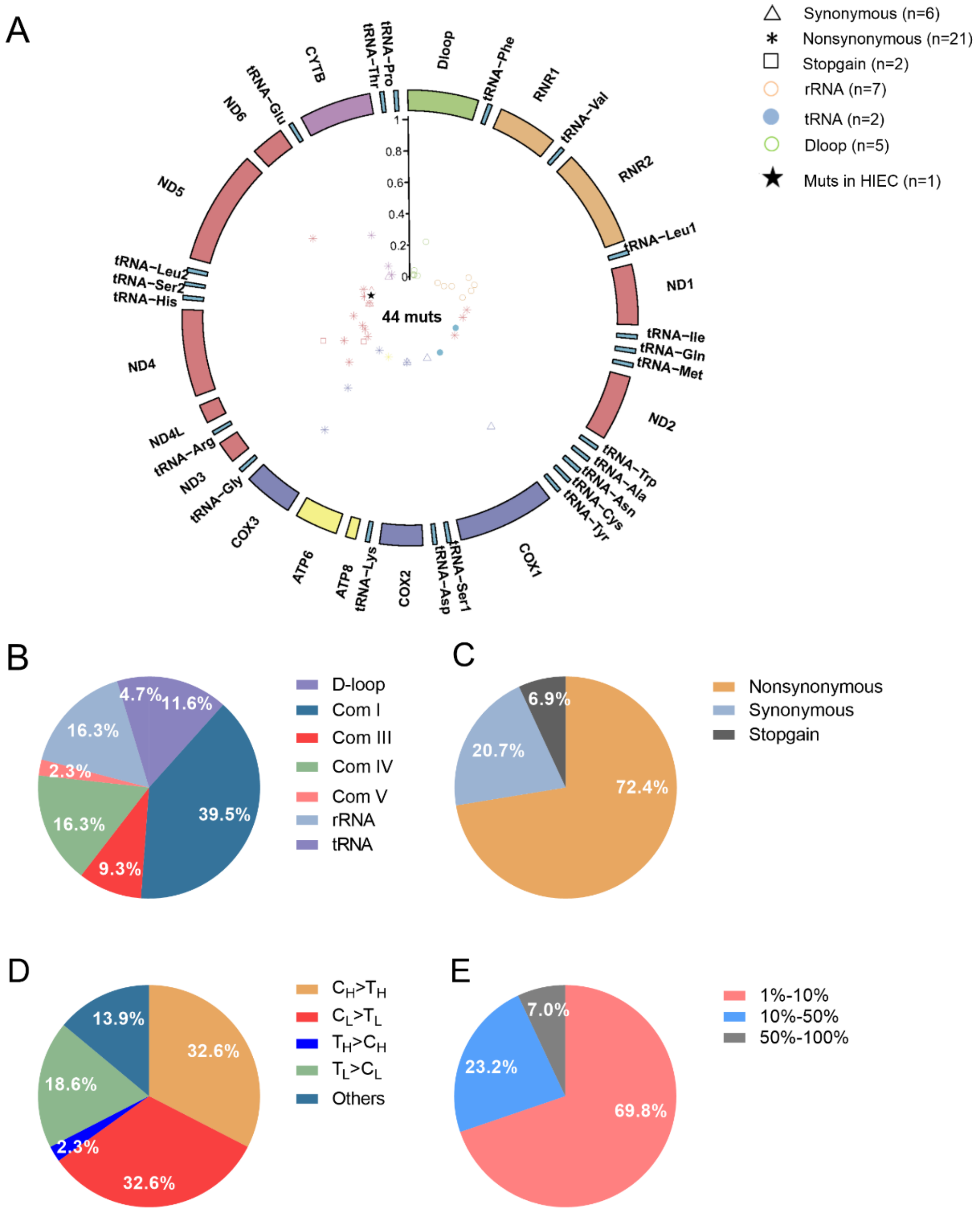

Supplementary Figure 2. Mutational pattern of mtDNA in 11 CRC and the HIEC control cell lines. (A) Circos plot showing the frequency and distribution of heteroplasmic mtDNA variants from 11 CRC (including DLD1 and HT29) and the HIEC control cell lines. **(B)** The percentage of heteroplasmic mtDNA variants in the functional regions of mtDNA, including D-loop, respiratory complexes (Com I, Com

III, Com IV, Com V), rRNA and tRNA genes in 11 CRC cell lines. **(C)** The percentage of synonymous, nonsynonymous, and stopgain heteroplasmic mtDNA variants in 11 CRC cell lines. **(D)** The percentage of base substitution types for heteroplasmic mtDNA variants in 11 CRC cell lines. H for heavy strand, L for light strand, and other for all transversion mutations. **(E)** The percentage of three different heteroplasmic levels for heteroplasmic mtDNA variants in 11 CRC cell lines. Muts, mtDNA mutations.

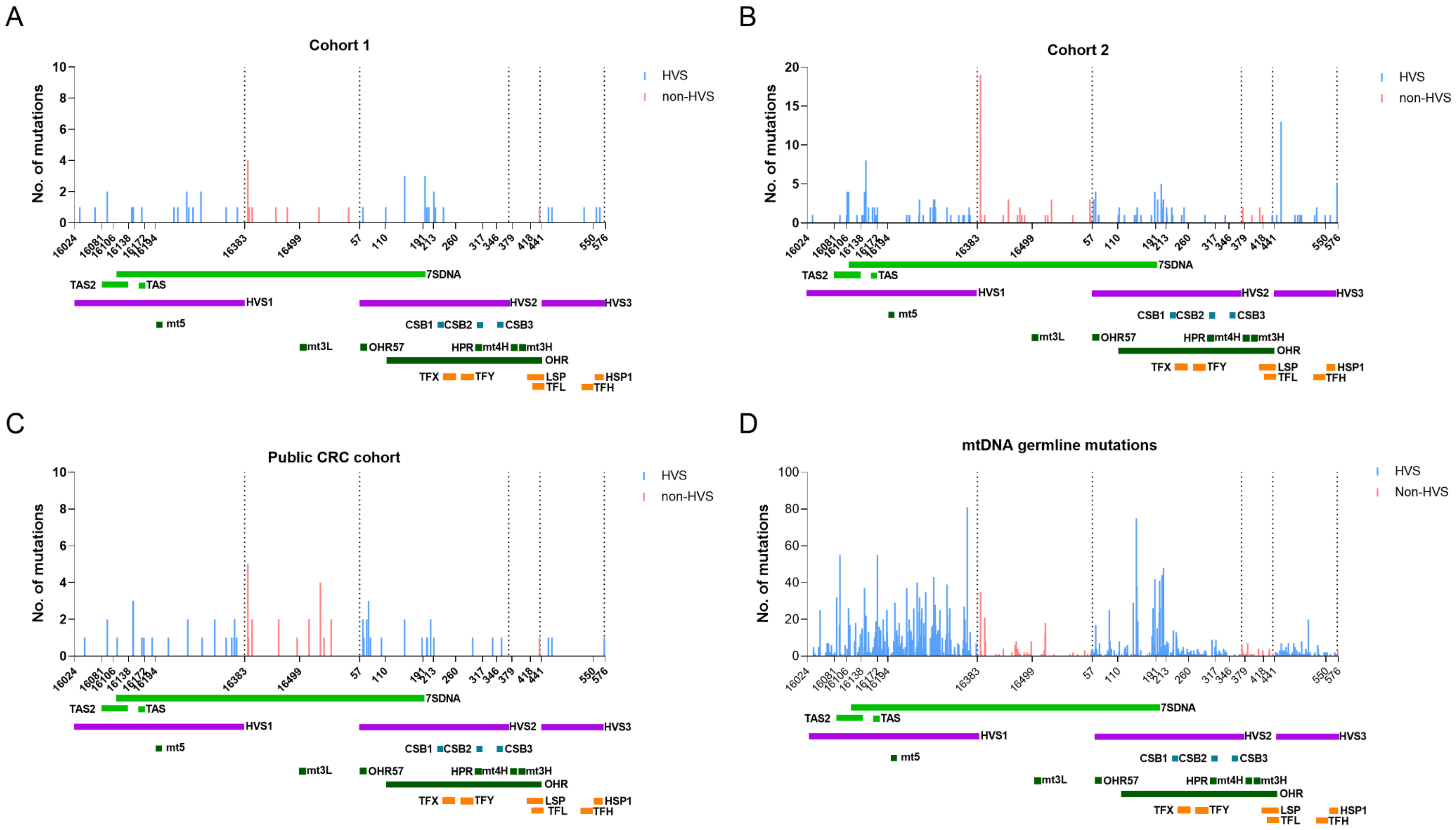

A
Cohort 1
B
Cohort 2
C
Public CRC cohort
D
mtDNA germline mutations
No. of mutations
HVS
non-HVS
Non-HVS
16024
16081
16106
16138
16172
16194
16383
16499
57
110
191
213
260
317
346
379
418
441
550
576
7SDNA
TAS2
TAS
HVS1
HVS2
HVS3
mt5
CSB1
CSB2
CSB3
mt3L
OHR57
HPR
mt4H
mt3H
OHR
TFX
TFY
LSP
TFL
HSP1
TFH

Supplementary Figure 3. Distribution of somatic mutations in three CRC cohorts **(A-C)** and germline mutations **(D)** in non-HVS and HVS of mtDNA control region (mtCTR). Functional units in mtCTR were also shown. HVS, hypervariable segment.

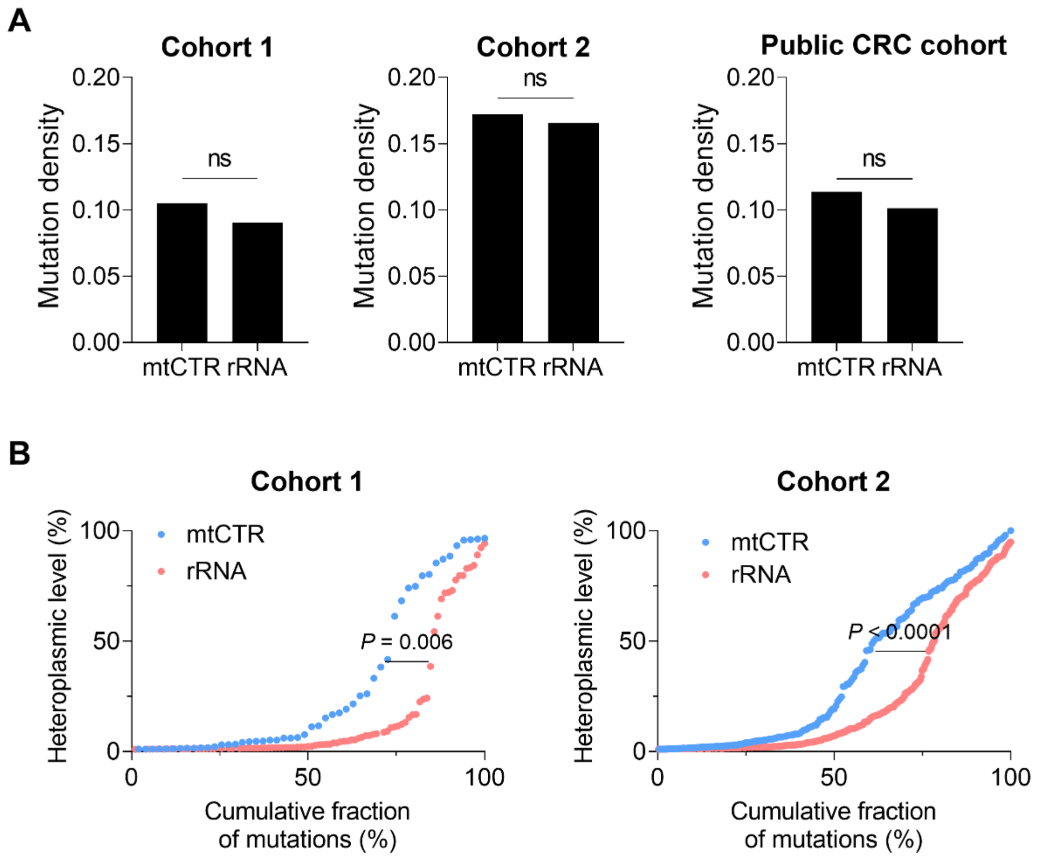

Supplementary Figure 4. mtDNA rRNA mutations are subjected to negative evolutionary selection in CRC. (A) The mutation density of mtDNA mutations in the mtDNA control region (mtCTR) and rRNA coding regions in three CRC cohorts. **(B)** Heteroplasmic level of accumulated mutations in mtCTR and rRNA coding regions in cohorts 1 and 2. Data were compared using Chi-square test **(A)** and Kolmogorov-Smirnov test **(B)**.

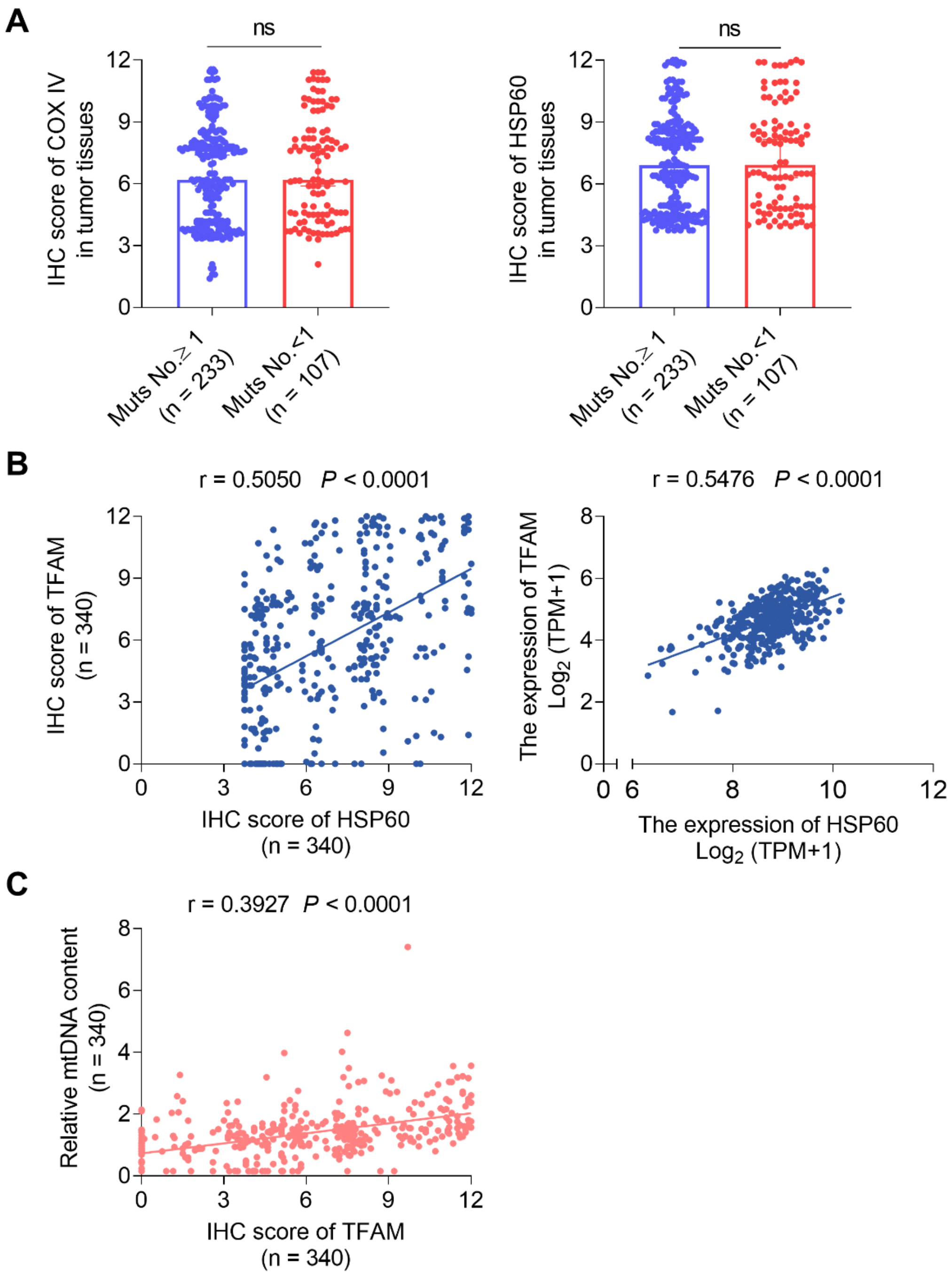

Supplementary Figure 5. Correlation between mtDNA mutations or TFAM expression and mitochondrial biogenesis biomarkers in CRC. (A) Comparison of HSP60 and COX IV expression between CRC tissues with high (Muts No. ≥ 1) or low (Muts No. < 1) mtDNA mutation load. **(B)** Spearman correlation analysis between the level of TFAM and HSP60 expression based on the IHC assay (n = 340) and

RNA-seq counts retrieved from TCGA. **(C)** Spearman correlation analysis between the relative mtDNA content and TFAM expression in CRC tissues (n = 340).

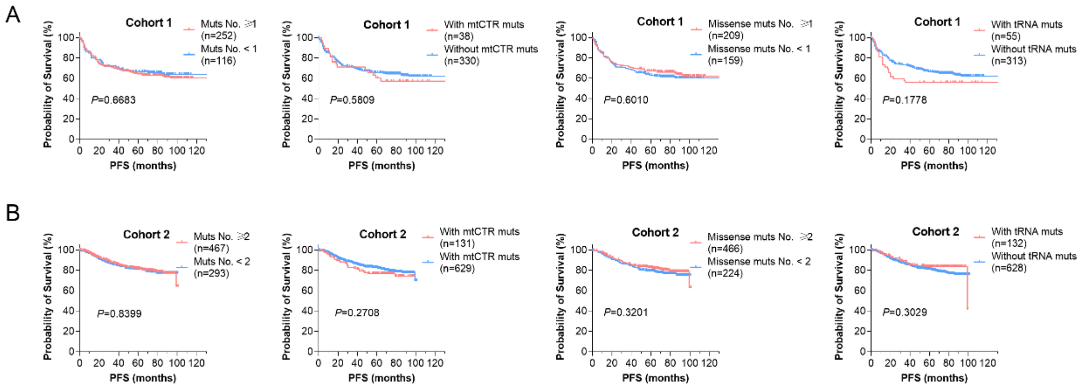

Supplementary Figure 6. mtDNA somatic mutations in CRC tissues are not associated with clinical progression. (A) Kaplan-Meier curve analysis of progression free survival (PFS) between patients with different number of mtDNA mutations, mtCTR mutations, missense mtCDR mutations, and tRNA mutations in CRC cohort 1. **(B)** Kaplan-Meier curve analysis of progression free survival (PFS) between patients with different number of mtDNA mutations, mtCTR mutations, missense mtCDR mutations, and tRNA mutations in CRC cohort 2. The median number of mtDNA mutations in cohorts 1 and 2 was used as threshold value to subgroup high or low number of mtDNA mutations, which was 1 in cohort 1 and 2 in cohort 2, respectively. *P* values were calculated using the log-rank tests. Muts, mtDNA mutations; mtCTR, mtDNA control region; mtCDR, mtDNA coding region.

Supplementary tables

Supplementary Table 1. Clinical characteristics of two private CRC cohorts.

**Characteristics**	**Cohort 1 (n = 432）**	**Cohort 2 (n = 1,015）**
**Age, median (range)**	59 (27-87)	59 (18-88)
**Gender, n (%)**		
Female	201 (46.5)	404 (39.8)
Male	231 (53.5)	611 (60.2)
**The primary site, n (%)**		
Colon	264 (61.1)	729 (71.8)
Rectum	168 (38.9)	286 (28.2)
**TNM stage, n (%)**		
I	41 (9.5)	91 (9.0)
II	209 (48.4)	384 (37.8)
III	154 (35.6)	287 (28.3)
IV	28 (6.5)	252 (24.8)
Unknown	0 (0)	1 (0.1)
**Metastasis status, n (%)**		
Metastasis	105 (24.3)	252(24.8)
No metastasis	272 (63.0)	763(75.2)
Unknown	55 (12.7)	0
**Histology, n (%)**		
Poor differentiated	50 (11.6)	222(21.9)
Moderately differentiated	316 (73.1)	731(72.0)
Well differentiated	43 (10.0)	6(0.6)
Mucinous	23 (5.3)	45(4.4)
Unknown	0 (0)	11(1.1)
**Death status, n (%)**		
Death	130 (30.1)	223（22.0）
Survival	238 (55.1)	792（78.0）
Unknown	64 (14.8)	0

**Relapse status, n (%)**		
Relapse	136 (31.5)	150 (14.8)
No relapse	232 (53.7)	610 (60.1)
Unknown	64 (14.8)	255 (25.1)

Supplementary Table 2. Summary of mtDNA sequencing data from two private CRC cohorts.

Sample source	Sample type	Sample number	Sequencing platform	Sequencing type	Average Q30	mtDNA coverage	Average depth (X)
Cohort 1	CRC	432	Illumina Hiseq	Capture	93.47%	100%	5,084 ± 2,357
	Para-CRC	432			93.78%	100%	5,200 ± 2,090
Cohort 2	CRC	1,015	Illumina Novaseq	WESplus*	95.55%	100%	9,668 ± 5,373
	Para-CRC	1,015			95.33%	100%	8,681 ± 5,211

Note: CRC: colorectal carcinoma tissue; Para-CRC: paired adjacent non-CRC colon/rectum tissue.

WESplus*: a capture-based next-generation sequencing product with improved sequencing depth that can detect mutations and copy-number alterations at the whole-exome level, which can also capture the whole genome sequence of mitochondria.

Supplementary Table 3. Summary of mtDNA somatic mutations in public CRC cohort.

Data source	mutation Cutoff	Sequencing platform	Sequencing type	Coverage region	Heteroplasmy level data	Samples (No.)	mtCDR mutations (No.)	mtCTR mutations (No.)
Nature Genetics 2020	≥ 1%	Illumina	WGS	Whole mtDNA	NA	60	148	42
PNAS 2012	≥ 5%	Illumina	WGS	Whole mtDNA	Available	129	146	3
Human mutation 2018	≥ 1%	Roche 454	WGS	Whole mtDNA	Available	100	122	26
Nature metabolism 2021	≥ 5%	Illumina	WES	Coding region	NA	556	650	NA
Total						845	1,066	71

Note: Considering the presence of duplicative samples, the somatic mutations summary of Nature genetics 2020 and PNAS 2012 were mixed with those from Nature metabolism 2021. If one sample was present in two or more datasets, all mutations in this sample were combined. mtCDR, mtDNA coding region; mtCTR, mtDNA control region.

Supplementary Table 4. Primary antibodies used for Western blot and immunohistochemistry (IHC).

Antibody	Company (Cat. No.)	Working concentration dilutions
HSP 60	Abcam (Ab46798)	WB: 1:1,000, IHC: 1:100
COX IV	Proteintech (#11242-1-AP)	WB: 1:1,000, IHC: 1:100
β-actin	Abcam (Ab179467)	WB: 1:3,000
Rabbit IgG	Cell Signaling (#2729)	WB: 1:10,000
Mouse IgG	Beyotime (A7028)	WB: 1:10,000
TFAM	Abcam (ab176558)	IHC: 1:200
